# Supplementary material for: Distinct Chemokine Receptor Expression Profiles in De Novo DLBCL, Transformed Follicular Lymphoma, Richter’s Trans-Formed DLBCL and Germinal Center B-Cells
Source: Int J Mol Sci. 2022 Jul 17;23(14):7874. doi: 10.3390/ijms23147874 (PMC9316992; doi:10.3390/ijms23147874)
Supplement: Supplementary file 1 [file ijms-23-07874-s001.zip › ijms-1825923-supplementary.pdf]

Supplementary Material

Table S1. **Nucleotide sequences of the forward primers, reverse primers, and probes for qPCR**

Gene	Primer forward (5′-3′)	Primer reverse (5′-3′)	Probe (5′-3′)
GAPDH	CTTCATTGACCTCAACTACATGTTTAC	TGACAAGCTTCCCGTTCTCA	[FAM]ATGGCAAATTCCATGGCACCGTCA[TAM]
PPIA	CCACCGTGTTCTTCGACATTG	ATCCTTTCTCTCCAGTGCTCAGA	[FAM]CCGCGTCTCCTTTGAGCTGTTTGC[TAM]
CCR1	CACGGACAAAGTCCCTTGGA	TGTGGTCGTGTCATAGTCCTCTGT	[FAM]TGGAGTTTCCATCCCGGCTTCTCTCT[TAM]
CCR2	GATGAATGGGAGTGAGGGATAGTG	GAGCCCTTTGCTTCACCTTTG	[FAM]TTTGTCCAGGCTCAGCCATGCTCA[TAM]
CCR3	CAACATCTACCTGCTCAACC	GCCAAAAACCCAGTTATGCC	[FAM]CCTGCTCTTCCTCGTCACCCTTCCATT TAM]
CCR4	TAATATTGCAAGGCAAAGACTATTCC	GCGATTTACTCCATCAGCCAGTA	[FAM]TGCAATTCCCTCTGGAGAAACCCATCA[TAM]
CCR5	GATTGATTTGCACAGCTCATCTG	TGTCATAGATTGGACTTGACACTTGA	[FAM]TCTCCCCGGGTGGAACAAGATGG[TAM]
CCR6	CAGAGCACTGCCTGAGAGTCAC	TGGTTGTAGAAAAAGGAGTGTATGGT	[FAM]AGGCAGTTCTCCAGGCTATTTGTACCGAT[TAM]
CCR7	GGGCACAGCCTTCCTGTG	CCACCACCAGCACGCTTT	[FAM]ACCGCCCAGAGAGCGTCATGGA[TAM]
CCR8	GAAGGAATTGGCAACACTGAAAC	ATCCATCAAGGCAGCGGGAC	[FAM]CCTCCAGAACAAAGGCTGTCACTAAGG[TAM]
CCR9	GACTTCACAAGCCCTATTCCTAACA	AAGTCAAGTGAAGTTGAAGTTAACGTAGTCT	[FAM]ACTATGGCTCTGAATCCACATCTTCCATGG[TAM]
CXCR1	CTCCTACTGTTGGACAC	ACATGTCCTCTTCAGTTTC	[FAM]CCGGTGCTTCAGTTAGATCAAACCA [TAM]
CXCR2	AGGTGTCCTACAGGTGAAAAG	AATCTTCAAAGCTGTCACTCTC	[FAM]CCAGCGACCCAGTCAGGATTTA[TAM]
CXCR3	CAGCCCAGCCATGGTCCTTG	GGAAGAGCTGAAGTTCTCCAG	[FAM[CTAAATGACGCCGAGGTTGCCGC[TAM]
CXCR4	CAATGACTTGTGGGTGGTTGTG	ATGCAATAGCAGGACAGGATGA	[FAM]CATGGTTGGCCTTATCCTGCCTGGTA[TAM]
CXCR5	CAGCCATGAACTACCCGCTAA	CCAATCTGTCCAGTTCCCAGA	[FAM]AGGTCCTCCAGGTTCTCGAGGTCCATT[TAM]
CXCR6	AGAGCAGCAGTGAAAACAAG	ACAAAAGTCAAGCCCCAAG	[FAM]TGGCACCACCAGGCACCTCAC[TAM]
CXCR7	CTACACGCTCTCCTTCATTTAC	TATTCACCCAGACCACCAC	[FAM]CGTCATCGGCATGATTGCCAACT[TAM]
CX3CR1	TGACTGGCAGATCCAGAGGTT	TTCTGTCACTGATTCAGGGAACTG	[FAM]AGTCCACGCCAGGCCTTCACCA[TAM]
XCR1	CCATCGTGGTGGCCTACTTC	CGCAGCTCCGGATGATCT	[FAM]TCTGCAGACGCTGTTTCGGACCC[TAM]

Table S2. List of antibodies used for immunohistochemistry

Antigen	Order number	Company	Clone	Dilution factor
**CCR1**	DF2710	Affinity Biosciences, Melbourne, Australia	Polyclonal	1:50
**CCR4**	bs-1168R	Bioss Inc, Massachusetts, USA	Polyclonal	1:100
**CCR5**	bs-2514R	Bioss Inc, Massachusetts, USA	Polyclonal	1:50
**CCR6**	66801-1-Ig	Proteintech, Chicago, USA	1C11A9	1:30
**CCR7**	MAB197	R&D Systems, Minneapolis, USA	150503	1:60
**CCR8**	PA5-34633	Thermo Fisher Scientific, Massachusetts, USA	Polyclonal	1:50
**CXCR2**	bs-1629R	Bioss Inc, Massachusetts, USA	Polyclonal	1:200
**CCL19**	MAB361	R&D Systems, Minneapolis, USA	54909	1:50
**CCL21**	MAB457	R&D Systems, Minneapolis, USA	59106	1:30
**MUM1**	M7259	Dako, Glostrup, Denmark	MUM1p	1:50
**CD10**	NCL-CD10–270	Novocastra, Leica Biosystems, Wetzlar, Germany	56C6	1:6
**BCL-6**	GA625	Aligent, California, USA	PG-B6p	Ready-to-use
**CD68**	GA609	Aligent, California, USA	KP1	Ready-to-use

Table S3. Antibodies used for multicolor-immunofluorescence staining

Specificity	Conjugate	Clone	Isotype	Manufacturer
**CD8**	PE	SK1	mouse IgG1, κ	BD
**CD3**	APC	SK7	mouse IgG1, κ	BD
**CD3**	PE	SK1	mouse IgG1, κ	BD
**CD3**	FITC	SK7	mouse IgG1, κ	BD
**CD4**	FITC	SK3	mouse IgG1, κ	BD
**Goat anti-mouse IgG1 (H+L)**	Alexa Fluor 546	N/A	goat IgG	Invitrogen
**Anti-FITC**	Alexa Fluor 488	N/A	goat IgG	Invitrogen
**Nuclei**	DAPI	N/A	N/A	Roche

FITC, fluorescein isothiocyanate; DAPI, 4′,6-diamidino-2-phenylindole; PE, R-phycoerythrin; APC, allophycocyanin; N/A, not applicable.

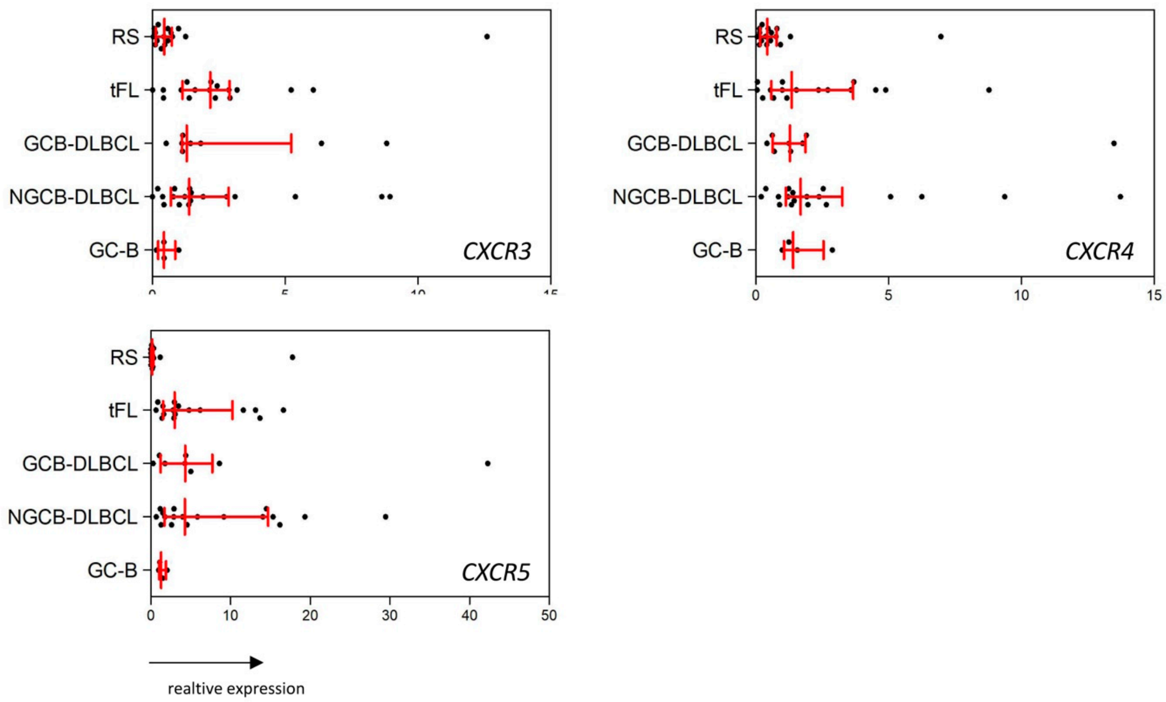

Figure S1. mRNA expression of CXC chemokine receptors in RS, tFL, GCB-DLBCL, NGCB-DLBCL, and as control in normal GC-B. Scatter plots show the mRNA expression of *CXCR3*, *CXCR4,* and *CXCR5* in RS, tFL, GCB-DLBCL, NGCB-DLBCL, and as controls in normal GC-B. GC-B denotes germinal center B cells, which were isolated from non-neoplastic tonsils. Values of gene expression are calculated as relative expression. Red lines indicate median and interquartile range.

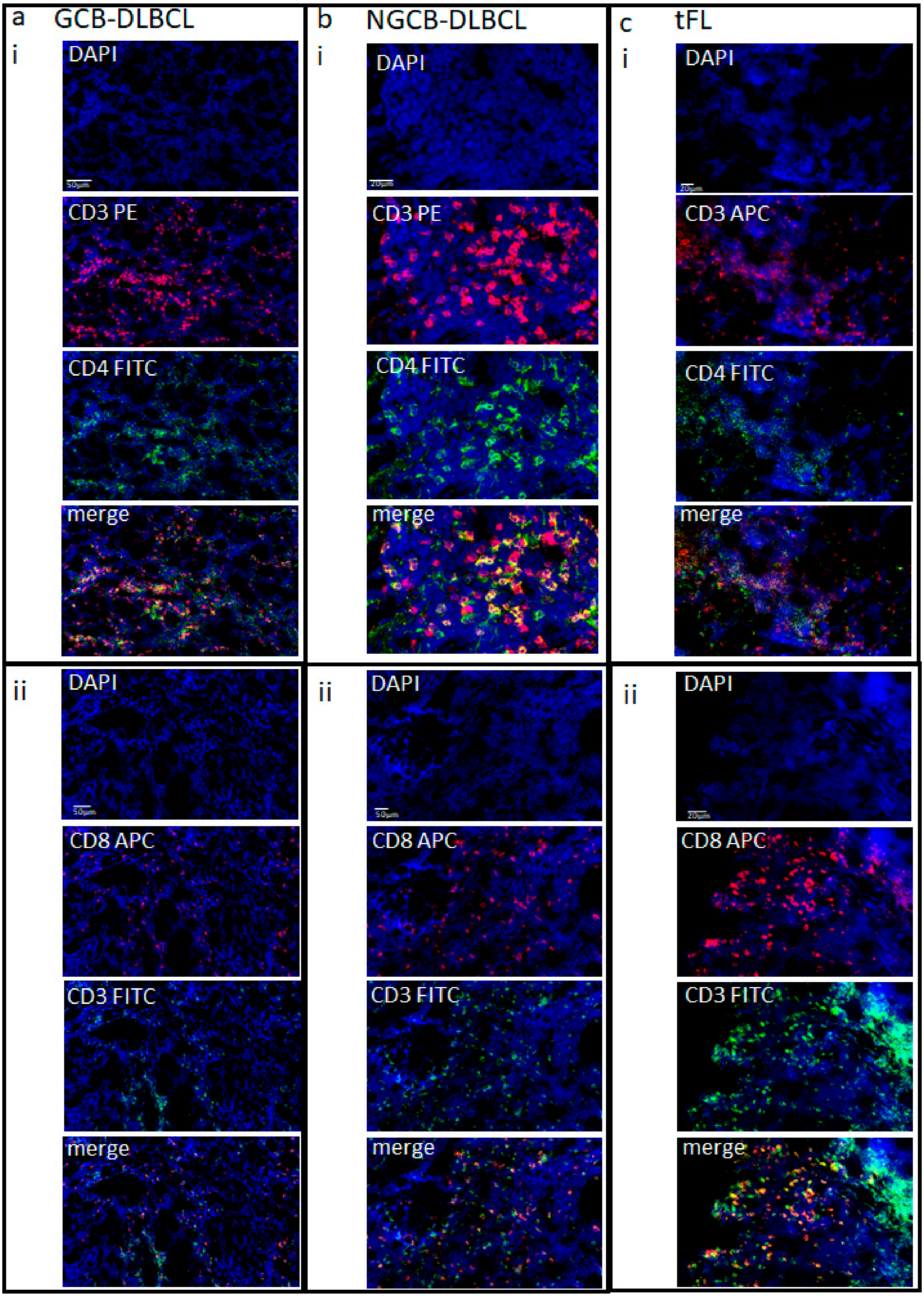

Figure S2. Representative images of IF multicolor stainings of helper T cells (CD3+CD4+) (i) and cytotoxic T cells (CD3+CD8+) (ii) merged with 49.6-diamidino-2-phenylindole (DAPI) in a GCB-DLBCL (a), NGCB-DLBCL (b) and tFL (c). Slides were scanned using a TissueFAXS imaging system (TissueGnostics GmbH) equipped with a Zeiss Axio Imager.Z1 microscope (Carl Zeiss Inc., Jena, Germany) with filters detecting DAPI, green fluorescent

protein, Cy3, and Cy5 fluorochromes. Images were taken with Zeiss LD Plan-Neofluar objectives (primary objective 320/0.4, ocular objective 310) at room temperature using a PCO PixelFly camera (Zeiss), exported from the TissueQuest software (TissueGnostics GmbH) as tiff images, and processed in Adobe Photoshop CS5 (Adobe Systems, San Jose, CA).

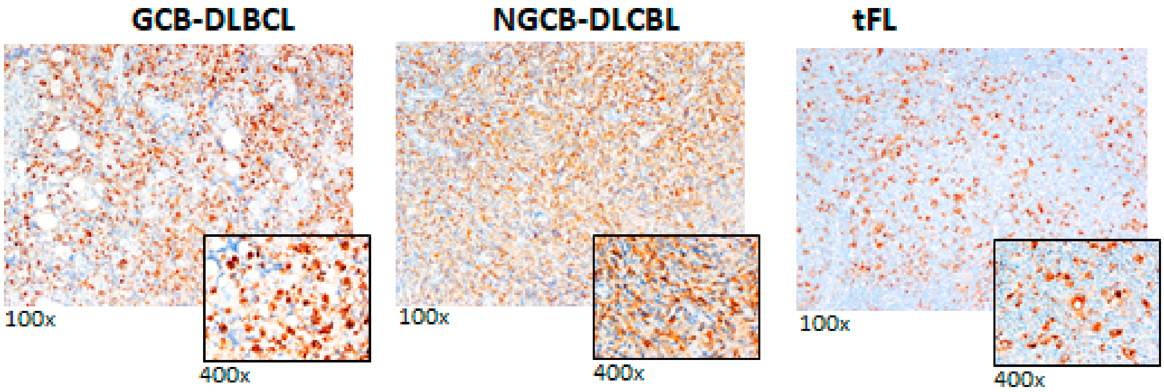

Figure S3. Immunohistochemistry of CD68 expression in GCB-DLBCL, NGCB-DLBCL and tFL. All images were captured using an Olympus BX51 microscope and an Olympus E-330 camera (magnification 100x for the overview and magnification 400x for the small picture).

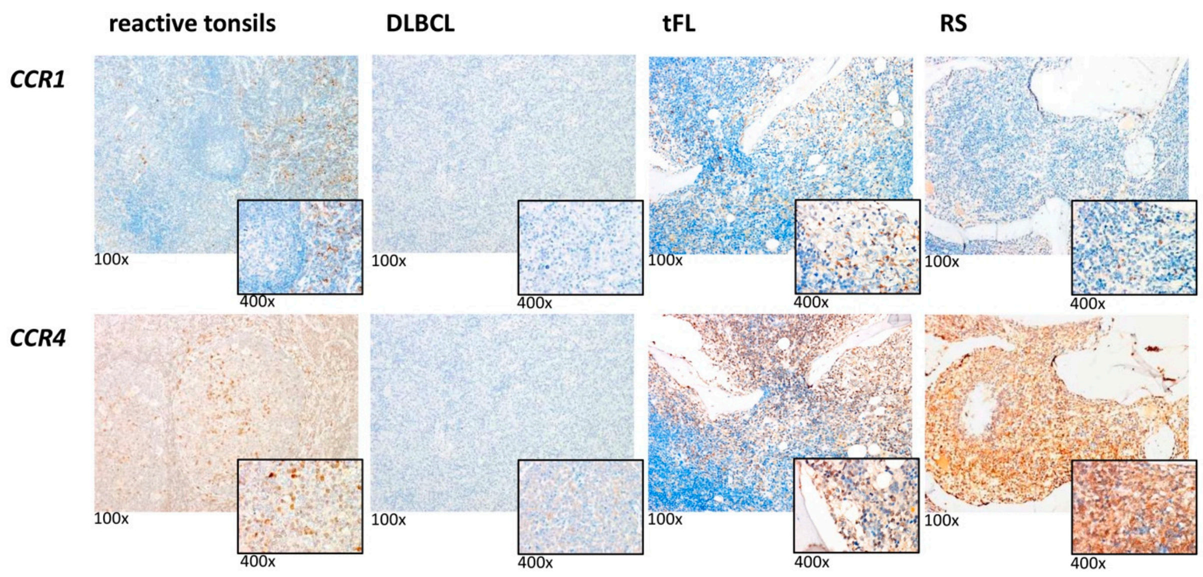

Figure S4: Immunohistochemistry of CCR1 and CCR4 expression in reactive tonsils, *de novo* DLBCL, tFL, and RS. All images were captured using an Olympus BX51 microscope and an Olympus E-330 camera (magnification 100x for the overview and magnification 400x for the small picture).
